# Challenging Conventional Wisdom: Early Treatment and Chronicity Outcomes in Acute Severe Hepatitis B

**DOI:** 10.7150/ijms.101261

**Published:** 2024-11-11

**Authors:** Dilara Turan Gökçe, Derya Arı, Melike Yakut, Burçak Kayhan, Emin Altıparmak, Adalet Altunsoy, Meral Akdoğan Kayhan

**Affiliations:** 1 Department of Gastroenterology, Sincan Education and Research Hospital, Ankara, Turkey.; 2 Ankara Bilkent City Hospital, Department of Gastroenterology, Ankara, Turkey.; 3 Ankara Bilkent City Hospital, Department of İnternal Medicine, Ankara, Turkey.; 4 Ankara, Turkey.; 5 Department of Infectious Disease and Clinical Microbiology, Ankara, Turkey.

**Keywords:** acute hepatitis B, antiviral treatment, chronicity

## Abstract

This study aimed to evaluate the clinical outcomes of patients diagnosed with acute severe hepatitis B (ASHB) who received early antiviral therapy compared to those who did not.

Patients diagnosed with acute hepatitis B between February 2019 and February 2023 at our hospital were retrospectively analyzed for admission characteristics, antiviral treatments, and serum HBsAg and anti-HBs levels over 3-6-12 months. Acute severe hepatitis B was defined as serum total bilirubin > 5 mg or INR > 1.5.

Of the 57 patients included, 26.3% (n=15) were female, and the median age was 40.2 (21-90) years. Within 48 hours of admission, 2 patients had concurrent diseases (3%) died. Two patients with concurrent HIV diagnosis were excluded. Treatment was initiated in 27 of 53 ASHB patients (entecavir/tenofovir: 24/3). One patient in the treatment group underwent liver transplantation due to fulminant hepatitis, and another patient died while on the waiting list. Long-term follow-up information for 3 patients in the untreated group was unavailable. The study continued with 25 treated and 23 untreated patients. No significant differences were observed in age, ALT levels, albumin, leukocyte, neutrophil, and platelet levels between the two groups (respectively; p = 0.57, p = 0.071, p = 0.187, p = 0.46, p = 0.94, p = 0.307). However, in the treated group, AST, total bilirubin, INR, and hospitalization duration were higher, and lymphopenia was more common. In the entire patient population, HBsAg seroclearance rates were 54% at 3 months (69% in treated vs. 34% in untreated; p = 0.127), 83.3% at 6 months (95% in treated vs. 74% in untreated; p = 0.218), and 100% at 12 months.

Early antiviral therapy did not show an association with chronicity in ASHB patients. Conducting randomized controlled studies with a larger patient population is necessary to provide a definitive conclusion on initiating early antiviral therapy. However, such studies pose ethical challenges.

## Introduction

Hepatitis B infection continues to be a significant public health concern globally. In 2021, there were 2,045 reported cases of acute hepatitis B in the USA 1. However, after adjusting for underascertainment and underreporting, the estimated number of infections in the USA was approximately 13,300 1. The incidence of acute hepatitis B has decreased following the implementation of the national vaccination program in Türkiye. However, there has been an observed increase in cases among the adult population. According to the Ministry of Health data, in 2017, the incidence rate of acute hepatitis B was recorded at 1.9 cases per 100,000 people 2.

The occurrence of acute hepatitis B represents a considerable public health risk due to its potential for severe outcomes and complications. The management of acute hepatitis B is crucial, as it can progress to acute liver failure in some cases 3. The clinical manifestations of acute hepatitis B can range from self-limited acute hepatitis to acute liver failure or chronic hepatitis B infection. Currently, there are no clear cut-off points for when treatment should be initiated, and indications for treatment can vary according to guidelines.

According to the European Association for the Study of the Liver (EASL) guidelines, severe acute hepatitis B is characterized by coagulopathy, typically defined as an international normalized ratio (INR) of 1.5 or higher. It may also present as a protracted course, with persistent symptoms or significant jaundice lasting for at least 4 weeks, or as signs of acute liver failure 4. The American Association for the Study of Liver Diseases (AASLD) recommends antiviral treatment for patients with acute hepatitis B who experience acute liver failure or have a prolonged, severe course. This recommendation is based on specific criteria, such as a total bilirubin level greater than 3 mg/dL (or direct bilirubin level greater than 1.5 mg/dL), an INR greater than 1.5, the presence of encephalopathy, or ascites 5.

In patients with acute hepatitis B, it is known that the rate of spontaneous seroclearance in adults is approximately 95% 6. Randomized controlled trials have explored antiviral treatment options, such as lamivudine, for acute hepatitis B 7,8. However, in contemporary practice, potent treatment options like entecavir and tenofovir disoproxil fumarate are considered the first line of therapy 4,5. Despite this, the application of these treatments remains limited and subject to debate. Consequently, our study aimed to investigate the development of seroclearance in groups receiving antiviral treatment versus those who did not and, to define indications of treatment initiation.

## Methods

### Patient selection

Patients over the age of 18 who were diagnosed with acute hepatitis B infection at Ankara Bilkent City Hospital between November 2019 and May 2023 were retrospectively included in the study if they had acute severe hepatitis B (ASHB). Based on guidelines and our clinical experience; patients with a serum total bilirubin > 5 mg/dl or INR > 1.5 or encephalopathy or ascites were considered to have acute severe hepatitis B. All patients admitted with acute hepatitis B were checked through the information system to ensure that they had negative HBsAg levels within the last 12 months, had no findings suggestive of chronic liver disease in their history and radiological findings, and were not receiving immunosuppressive therapy. Additionally, it was confirmed that all patients with positive HBsAg had high-titer, positive anti-HBc IgM and high HBV DNA viral load. It was confirmed that all patients were tested for other causes of acute hepatitis, including Hepatitis A virus, Hepatitis C virus, and TORCH infections. Each patient was also questioned for drug toxicity and herbal use. Every patient was tested for Hepatitis D virus. Due to the retrospective nature of this study, there is no standardization for initiating treatment. At our hospital, patients with acute severe hepatitis B are monitored according to clinical follow-up protocols, with the decision to start treatment made by at least two experts (hepatologists or infectious disease specialists) based on disease progression. Additionally, since antiviral treatment for acute hepatitis B is not covered by insurance under Ministry of Health guidelines, a report for each patient's clinical status is submitted to the Ministry's pharmaceutical department to obtain treatment approval. Patients who received antiviral treatment until seroclearance developed were included in the study, while those in the treatment group who discontinued the medication or follow-up were excluded.

### Study groups

Among these patients, those in the treatment group were defined as those who received potent antiviral treatment in the early period (within the first 7 days); the non-treatment group consisted of those who did not receive antiviral treatment during follow-up due to ASBH. Only those who had been followed up for 12 months or more were included in the study.

### Study endpoints

The initial values at the time of patient admission, clinical and demographic characteristics, morbidity and mortality, and seroclearance and seroconversion information at 3, 6, and 12 months were meticulously recorded. The primary endpoint of the study was the development of HBsAg seroclearance, indicating the loss of hepatitis B surface antigen from the blood. Secondary endpoints included the necessity for liver transplantation and overall mortality rates. These endpoints were chosen to comprehensively evaluate the efficacy of the treatment and its impact on patient outcomes over the study period.

### Statistical analyses

The statistical analyses were performed with IBM SPSS Statistics for Windows, Version 26.0 software (IBM Corp., Armonk, NY, USA). In our analyses, we assessed whether continuous variables met the assumption of normal distribution. For continuous variables that adhered to the normal distribution assumption, independent samples t-tests were conducted to compare groups. For continuous variables that did not meet the normal distribution assumption, the Mann-Whitney U test was utilized. Comparisons of categorical variables were performed using Fisher's Exact Test. These statistical analysis methods are elaborately explained in the statistical analysis section of our research.

### Ethical approval

The ethics committee approved the study of Ankara Bilkent City Hospital (Approval number:E2-23-4744). The study was conducted in accordance with good clinical practice principles and the Declaration of Helsinki.

## Results

### All populations

**Figure 1:**
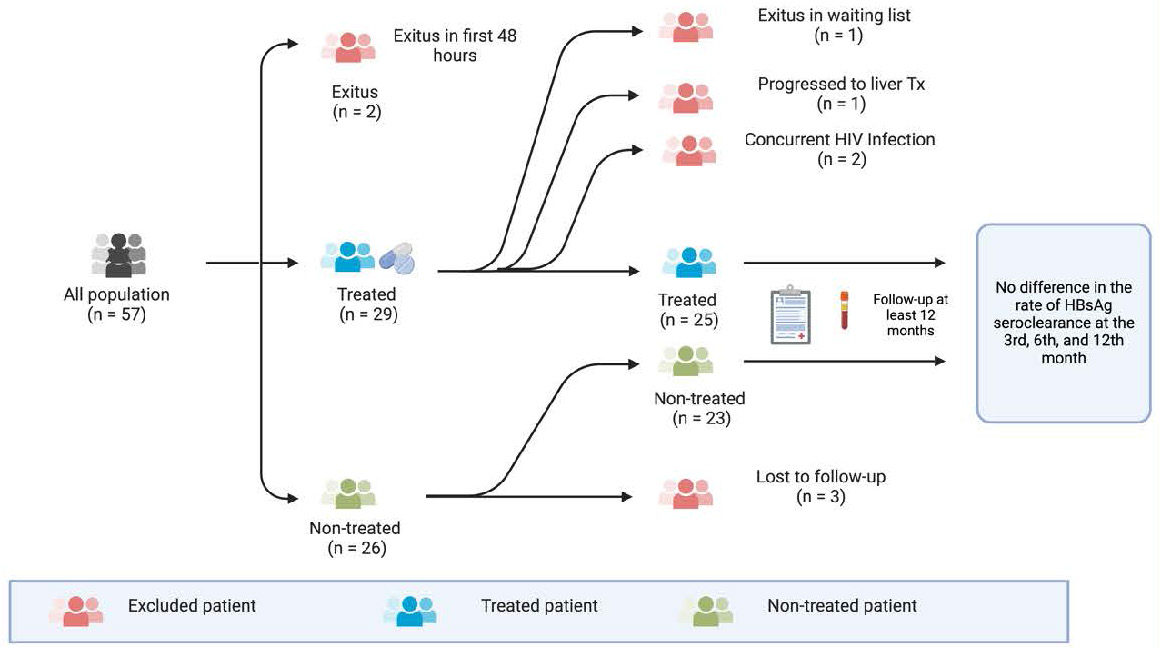
The visual chart of the patients evaluated in the study.

The long-term follow-up of the patients included in the study is shown in the flowchart in Figure 1**.** Among the 57 patients included in the study, 26.3% (n=15) were female, with a median age of 40.2 years (range 21-90 years). Upon analyzing the distribution of diagnoses across seasons for the entire patient group, spring emerged as the most common season for diagnosis (n=17), followed by winter (n=14), summer (n=13), and autumn (n=9), respectively. However, no statistically significant difference was observed between the seasons and the frequency of diagnoses (p=0.423). At the time of presentation, 45 patients (79%) were observed to be HbeAg positive.

### Mortality and liver transplantation

Upon evaluating all patients, mortality within the first 90 days post-diagnosis was observed in 3 patients (5.2%). Within 48 hours of admission, 2 patients (3%) succumbed to their conditions. One of them (patient 1), who was 34 years old, had a history of mushroom toxicity. The other patient (patient 2), who was 52, had severe COVID-19 pneumonia. Both of them had high levels of HBV DNA and serum levels of anti-HBc IgM, and they exhibited features of acute liver failure. The third patient (patient 3), who was 63 years old, passed away on the 54th day while on the waiting-list for liver transplantation, as he had no living liver donor candidate. This patient had a history of diabetes mellitus, hypertension, and obesity (BMI: 32 kg/m^2^). Additionally, one patient (patient 4), a 46-year-old woman, underwent cadaveric liver transplantation due to fulminant hepatitis. A common characteristic among patients with fatal outcomes or those who underwent transplantation was a serum total bilirubin level >20 mg/dL or an INR >2 at admission.

### Concurrent disease & unfollow patient group

Treatment was initiated in 27 out of 53 ASHB patients (entecavir/tenofovir disproxyl fumarate: 24/3). Two patients with a concurrent HIV diagnosis were excluded from the study. Long-term follow-up information was unavailable for 3 patients in the untreated group.

### Long-term follow-up

Analyses for the study were conducted with 48 patients who had been followed up for 12 months or longer. In the treatment-initiated group, the shortest duration until HBsAg seroclearance was observed, with the longest duration being 12 months of antiviral therapy. Quantitative changes are indicated in Table 1. The comparison of age between patients given antiviral treatment and those not given antiviral treatment showed no significant difference (median age for patients given antiviral: 41 years vs. median age for patients not given antiviral: 40 years; p = .571). Significant differences were observed in AST levels and total bilirubin levels (p = .005). Peak bilirubin levels also showed significant differences (p = .001). Peak INR values differed significantly between the groups (p = .012). No significant differences were found in leukocyte, hemoglobin, platelet counts, and albumin levels between the two groups (p > .05). The median HBV DNA levels were compared between patients who received treatment and those who did not. The treatment group had a median HBV DNA level of 2,091,840 IU/ml (min-max: 15,532 IU/ml - 243,369,633 IU/ml), while the non-treatment group had a median HBV DNA level of 444,005 IU/ml (min-max: 34,552 IU/ml - 32,815,459 IU/ml) (p=.105).

A significant difference was observed in D-dimer levels (p = .049), but not in fibrinogen levels (p > .05). Significant differences were noted in lymphocyte counts (p = .024). The duration of hospital stay showed significant differences (p = .018).

**Figure 2:**
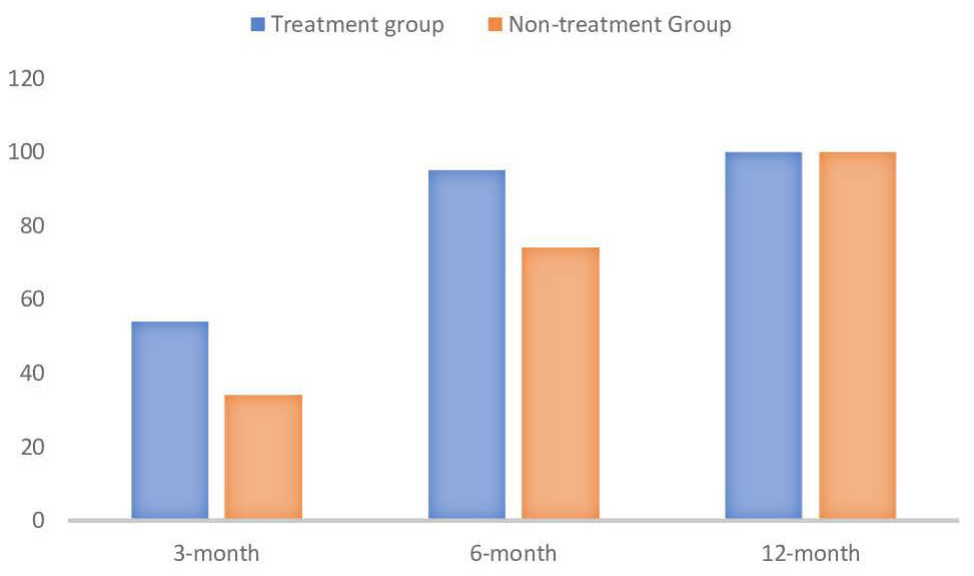
The percentage rate of HbsAg seroclearence (p values in order are p=0.127, p= 0.289 and NA) (created with BioRender.com).

The analysis of gender distribution among patients showed no significant difference between males and females in terms of receiving antiviral treatment (p = .815) (Table-2 & Figure-2). Gender distribution did not significantly influence the likelihood of receiving antiviral treatment.

When examining the primary endpoint of seroclearance (Table-2), it was observed that at the 3-month mark, there was no significant difference in HbsAg clearance between patients who received antiviral treatment and those who did not (p = .127). Specifically, 18 patients who received treatment achieved seroclearance, compared to 8 patients who did not receive treatment. Conversely, 8 patients treated with antivirals did not achieve seroclearance, compared to 10 untreated patients. Similarly, at the 6-month evaluation, the difference in HbsAg clearance between the two groups was not statistically significant (p = .289). By the 12th month, seroclearance was observed in all patients. Serconversion was seen in all patients who achieved seroclearance, with 2 patients showing seroclearance at the visit before last, 8 patients at the visit after seroclearance, and 38 patients at the same visit as seroclearance. These findings suggest that, within the timeframe of our study, antiviral treatment did not significantly impact the rate of HbsAg clearance at the 3-month and 6-month marks.

Due to the retrospective nature of the study, there was no standardization in the timing of HBV DNA level measurements. In 86% of the patients, HBV DNA levels were measured between 3 to 6 months of follow-up, and in 82% of the patients, serum HBV DNA levels were undetectable.

## Discussion

In our study, data from 57 patients presenting with acute severe hepatitis B were analyzed. The 90-day mortality rate in our study population was observed to be 5.2%. Among the entire population, one patient on the transplant list passed away, and one patient progressed to transplantation. Of the 48 patients who were followed for more than 12 months and observed retrospectively, 25 received treatment while 23 were monitored without treatment. No significant statistical difference was observed in the development of HbsAg seroclearance between patients who received treatment and those who did not within 3-6 months; however, HbsAg seroclearance was observed in all patients at the 12th month.

In a study conducted in Italy involving 18,460 cases of acute hepatitis B, the mortality rate was identified as 0.1% 9. This rate represents the mortality across all cases of acute hepatitis B, ranging from mild to severe. However, our patient group included only those defined as having severe acute hepatitis B, and the mortality rate in this group was determined to be 5.2%. Although this mortality rate may seem high, it is important to note that one of the deaths was associated with concurrent COVID-19, and another patient suffered from simultaneous mushroom intoxication. Furthermore, one patient, representing 1.7% of the study population, developed acute liver failure following severe acute hepatitis B and subsequently died due to the unavailability of a suitable donor.

The mortality rate associated with acute hepatitis was 3 per 100,000 population in the 1980s, but it decreased to 0.16 by the year 2019 in Türkiye 10. Along with Türkiye, this rate has been progressively declining worldwide 10. Acute hepatitis B has played a significant role in the mortality related to acute viral hepatitis. The reduction in mortality over the years can be attributed significantly to the World Health Organization's hepatitis B vaccination program and the impact of potent antiviral agents. In Turkey, a routine vaccination program was established for children born after 1998. However, the average age in our study is in the 5th decade, and this age group was not included in the routine vaccination program.

In our study, out of 57 patients, two were co-infected with HIV, presenting a contrast to the cohort described where 5443 HIV+ patients were monitored from 2000 to 2018. In that larger study, 18 developed acute hepatitis B (AHB), with a notably low global incidence of 0.02 per 100 patient-years across the entire population, and 0.06 per 100 patient-years among those who were anti-HBc negative 11. This incidence also showed a statistically significant decrease over the years. Additionally, in our study, it is noted that one of the two patients still tested positive for the HBsAg antigen at 15 months. In comparison, our smaller sample size and the presence of only two HIV co-infected patients limit the direct comparability but underscore the importance of monitoring and managing HIV-infected individuals for hepatitis B, especially considering the potential for chronic hepatitis development, as observed in 11% of the cases in the larger study. This comparison highlights the need for targeted preventive measures and treatment strategies in populations at higher risk of co-infection.

We observed a 6-month HBs Ag positivity rate of 14.6% among all groups and 26 % among non-treatment group, significantly higher than the 4.6% reported in another study involving 240 acute hepatitis B patients 11. Additionally, another local study reported a mortality rate of 2.5% and a one-year continuation of serum HBs Ag positivity at 10.6% 12. These figures suggest a potential decline in HBs Ag positivity over time, possibly due to natural disease progression or therapeutic interventions. The variations in HBs Ag positivity rates and outcomes highlight the need for continuous monitoring and tailored therapeutic strategies to improve treatment protocols and public health policies in managing hepatitis B.

In our study, 48 patients completed the study, of which 25 received antiviral treatment (entecavir: 22 & tenofovir disoproxil fumarate: 3). Previously, randomized controlled trials have been conducted to understand the impact of antiviral treatment on the course of the disease. In a study comparing the efficacy of Lamivudine versus placebo in treating acute hepatitis B, it was found that after 12 months, the seroclearance rates were significantly high in both groups, with 93.5% in the Lamivudine group and an even higher rate of 96.7% in the placebo group 6. This study has shown that in the treatment of acute hepatitis B, the use of Lamivudine did not create a clinically or biochemically significant difference compared to the placebo group. Early treatment with lamivudine in patients with severe acute hepatitis B significantly reduced HBV DNA levels and improved clinical outcomes and mortality, although it resulted in a lower rate of seroconversion 7. Lamivudine may improve outcomes in severe acute hepatitis B infection, but the small number of patients involved in the study limits definitive conclusions 13. In a comparison of antiviral therapies, entecavir (0.5 mg/day) showed the highest rate of HBsAg seroclearance at 24 weeks (59%), followed by placebo (39%) and lamivudine (100 mg/day) at 23%, with lamivudine also leading to significant reductions in viral load but lower seroconversion rates compared to controls 14. In a study of 32 patients with acute liver failure due to acute hepatitis B, all of whom received antiviral treatment (3 with lamivudine, 21 with entecavir, and 8 with tenofovir), 29 patients completed the process without transplantation, and 24 remained under long-term follow-up, with all achieving 100% seroclearance by day 108 at the latest 15. All 24 patients with severe acute hepatitis B were treated with potent antivirals, and it has been concluded that early treatment with entecavir or tenofovir disoproxil fumarate prevents the need for liver transplantation and the consideration of living donors 16. In our study, two patients died within the first 48 hours of presentation due to accompanying comorbidities. From the treated group, one patient progressed to transplantation, while another died while on the waiting list. Due to the retrospective nature of the study, it is not appropriate to compare progression to transplantation and mortality data with the group that did not receive medication. To obtain this information, a randomized controlled trial is needed, but due to the mortality risk associated with severe acute hepatitis B, randomized trials are not always feasible.

During the follow-up, every patient who was followed for at least 12 months developed seroclearance, which is significantly higher than expected. Among the six patients included in the study who were HBsAg positive at the 6-month mark, HBV DNA levels were available for three patients. Two of these patients had undetectable HBV DNA levels, while one patient experienced a drop in HBV DNA by more than 2 logs. However, three patients who dropped out of the follow-up were in the group that did not receive antiviral treatment, and we do not have data on whether these patients progressed to chronic hepatitis B infection. Since no patients in our study group developed long-term seropositiviy factors such as age, severity of the disease, and antiviral treatment that might predict chronic progression could not be evaluated. Nevertheless, since no chronic progression was observed in either the treated or untreated groups, it can be said that receiving antiviral treatment does not increase the risk of progression to long term seropositivity and is similar in this regard to being untreated.

The most significant limitation of this study is its retrospective design, which precluded a clear evaluation of bilirubin and INR levels in both the treated and untreated groups. However, it is acknowledged that designing a prospective randomized study poses ethical challenges. The study included patients who were followed for at least 12 months and continued treatment until HBsAg seroclearance. However, the intervals of follow-up for these patients were irregular, and there were no definitive data provided regarding the normalization of AST-ALT levels. Due to the retrospective nature of the study, information on the transmission route of the patients could not be obtained; however, this was not considered an important factor that would affect chronicity.

In conclusion, patients presenting with acute liver failure have a very high risk of mortality, and bilirubin levels alone are not sufficient to predict this outcome. This study is one of the most significant studies comparing potent antivirals with the non-treatment group over the long term. The treatment initiated in these patients did not show any effect on HBsAg seroclearance or seroconversion, and no serious side effects were observed in any of the patients. In light of these data, the decision to start treatment should be made on an individual patient basis. Current guideline recommendations are not sufficient to determine the criteria for initiating treatment in acute hepatitis B, and further research may be needed to explore the long-term effects of antiviral treatment on HbsAg clearance and to investigate other factors that may influence treatment outcomes.

## Figures and Tables

**Table 1:** The Numerical Findings of the Entire Study Population

Parameter	All Patients	Patients Given Antiviral (n:25)	Patients Not Given Antiviral (n: 23)	P-value
Age, year	40 (21-90)	41 (29-79)	40 (22-91)	.571
AST (U/L)	1363 (159-4563)	1152 (186-3566)	1283(159-3566)	.021
ALT (U/L)	1847 (32-5533)	2115 (32-5533)	1618 (233-3860)	.071
GGT (U/L)	137 (27-912)	128 (27-418)	179 (129-962)	.225
ALP (U/L)	205 (83-92)	202 (83-368)	221 (129-962)	.628
HBV DNA (IU/ml)	1,267,922 (15,532-243,369,633)	2,091,840 (15,532 - 243,369,633)	444,005 (34,552-32,815,459)	.105
Initial Total Bilirubin (mg/dl)	11.4 (4.1-32)	13 (4.1-32)	8.55 (4.3-18.4)	.005
Peak Bilirubin(mg/dl)	12.2 (5.0-32)	14.6 (5.1-32)	10.5 (5.0-18.4)	.001
Peak INR	1.36 (1-3.6)	1.49 (1-3.6)	1.3 (1-2)	.012
Leukocyte (x10^9^)	7050 (±1605)	6990 (±1669)	6669 (±1563)	.740
Hemoglobin (g/dL)	14.2 (9.2-16.2)	14.5 (10.6-16.1)	14 (9.2-16.2)	.464
Platelet (x10^9^)	225 (127-445)	223 (127-420)	234 (128-445)	.307
Neutrophil (x10^6^/L)	3800(1382-2090)	3960 (2090-6840)	3475 (2400-6840)	.945
Lymphocyte (x10^6^/L)	1540 (620-2840)	1355 (620-2480)	1670 (1220-2840)	.024
Albumin (g/L)	36.4 (±4.94)	35 (±4.57)	37.5 (±5.28)	.187
D-dimer (mcg/ml)	0.925 (0.19-8.34)	1.48 (0.19-8.34)	0.865 (0.19-3.18)	.049
Fibrinogen (mg/L)	2.42 (±0.655)	2.49 (±0.739)	2.31 (±0.512)	.328
Hospital Stay Duration (days)	11 (0-53)	14.5 (4-53)	9 (0-23)	.018
D-dimer/Platelet Ratio	0 (0-0.06)	0.01 (0-0.06)	0 (0-0.02)	.083
NLR		2.81 (0-4.88)	2.14 (0-4.54)	.17

Note: Data are summarised according to distributional characteristics, normally distributed data are shown as mean (±standard deviation) and non-normally distributed data are shown as median (min-max). *The laboratory values for the treatment group are shown at the time when the medication was initiated.

**Table 2:** Comparison of HbsAg Clearance and Categoric Variables Between Patients Receiving and Not Receiving Antiviral Treatment

Outcome	Treatment Group (n,%)	Non-Treatment Group (n,%)	P-value
3-month HbsAg Clearance			
- Seroclearance	14 (54)	8 (34)	.127
6-month HbsAg Clearance			
- Seroclearance	24 (95)	17 (74)	.289
12-month HbsAg Clearance			
- Seroclearance	25 (100)	23 (100)	NA
Gender (n:48)			
- Female	5	4	.815
- Male	20	19	

*NA: Not Applicable
